# ALK1 Gene Rearranged Pulmonary Sarcomatoid Carcinoma Masquerading as Tuberculosis in a Young Male

**DOI:** 10.5146/tjpath.2020.01481

**Published:** 2021-01-15

**Authors:** Ayushi Sahay, Rajiv Kumar, Amit Janu, Kumar Prabhash

**Affiliations:** Department of Pathology, Tata Memorial Center, Homi Bhabha National Institute (HBNI), Parel, Mumbai, India; Department of Radiology, Tata Memorial Center, Homi Bhabha National Institute (HBNI), Parel, Mumbai, India; Department of Medical Oncology, Tata Memorial Center, Homi Bhabha National Institute (HBNI), Parel, Mumbai, India

**Keywords:** Pulmonary sarcomatoid carcinoma, Pulmonary tuberculosis, ALK gene rearrangement, Lung cancer, Crizotinib

## Abstract

Pulmonary sarcomatoid carcinoma is rare, with limited treatment options and poor prognosis. In contrast to other non small cell lung carcinomas, not much is known about its molecular biology. In an endemic country like India, lung cancer is often masked by tuberculosis and presents in advanced stages. We report here an unusual case of pulmonary sarcomatoid carcinoma, in a young non-smoker male, who had co-existent tuberculosis masking and delaying the diagnosis of malignancy. On molecular study, the tumor showed ALK gene rearrangement, both by immunohistochemistry and fluorescence in-situ hybridization, which has been reported only twice previously. Presence of ALK gene rearrangements in sarcomatoid carcinoma has significant therapeutic implications and potential for altering the prognosis of this fatal disease. Hence we recommend performing ALK gene rearrangement analysis in all cases of sarcomatoid lung carcinomas. The report discusses the diagnostic approach and provides insight into the molecular pathogenesis of this exceedingly rare malignancy.

## INTRODUCTION

Pulmonary sarcomatoid carcinoma (PSC) is a rare, clinically aggressive subtype of non-small-cell lung carcinoma (NSCLC), that accounts for <1% of all lung cancers (1,2). In contrast to NSCLC, especially Adenocarcinoma, wherein discovery of targetable therapy (e.g. for EGFR, ALK1 and ROS1) has resulted in remarkable impact in the therapeutic outcomes, the genetic abnormalities in PSC are largely poorly defined. Hence, very limited targeted therapeutic options are available, till date (3–5).

We herein report a unique case of ALK1 gene rearranged PSC in a young male, which was masked by co-existent tuberculosis. ALK1 gene rearrangements are rarely described in PSC and till date only a handful of case reports have been published in the literature.

## CASE REPORT

A 33-year-old, non-smoker male presented with history of low grade fever and weight loss for the last year. He was initially evaluated in some other hospital. A large necrotic left para-tracheal mediastinal lymphadenopathy, near the arch of the aorta was detected by computerized tomography (CT) scan. He was empirically started on anti-Koch’s therapy (AKT) and showed partial initial response to the therapy as his fever subsided with treatment, the nutritional status improved, and the size of the lymph nodes were reduced on the follow-up CT scans. Seven months later, a cystic collection developed in the supraclavicular region, and fine needle aspiration cytology and histopathology showed necrotising granulomatous inflammation, which was positive for acid fast bacilli by the ZN stain. A TB Genexpert analysis confirmed Mycobacterium tuberculosis and hence, AKT was continued.

In addition, he developed a gradually progressing painful swelling on the left side of the chest wall over a period of four months. CT scan reported an infective collection with osteomyelitis involving the 7th and 8th ribs. He underwent drainage of the collection, with excision of the underlying 7th, 8th and 9th ribs. Surprisingly, histopathological examination showed a high grade malignant tumor. The patient was then referred to our cancer centre for further management. On histopathology review, a poorly differentiated malignant tumor composed of large spindled to epithelioid cells with areas of necrosis was seen. No definitive glandular or squamoid differentiation was noted. Frequent mitoses (18-20/10HPF) were noted. The underlying ribs as well as resection margins were also involved by the tumour. The differential diagnosis based on histopathology ranged from malignant mesothelioma, epithelioid sarcoma, malignant melanoma to poorly differentiated sarcomatoid carcinoma. Accordingly, immunohistochemistry (IHC) was performed. The tumour was strongly and diffusely positive for AE1/AE3, and CK7, while negative for calretinin, WT1, and D2-40 (podoplanin) (mesothelial markers); S100 protein and Melan A (melanoma markers), and CD34 (epithelioid sarcoma markers). The tumor cells were also negative for other epithelial (CK20, TTF1, Hep-Par1, PAX8) and mesenchymal markers (CD31, Desmin, SMA, S100). INI1 was retained in the tumor cells. Hence, possibility of metastatic poorly differentiated carcinoma with sarcomatoid differentiation and involving the chest wall was suggested and radiological correlation for the possible primary site was requested. Serum tumor markers for CEA (0.76 ng/ml) were determined and were within normal limits.

**Figure 1:**
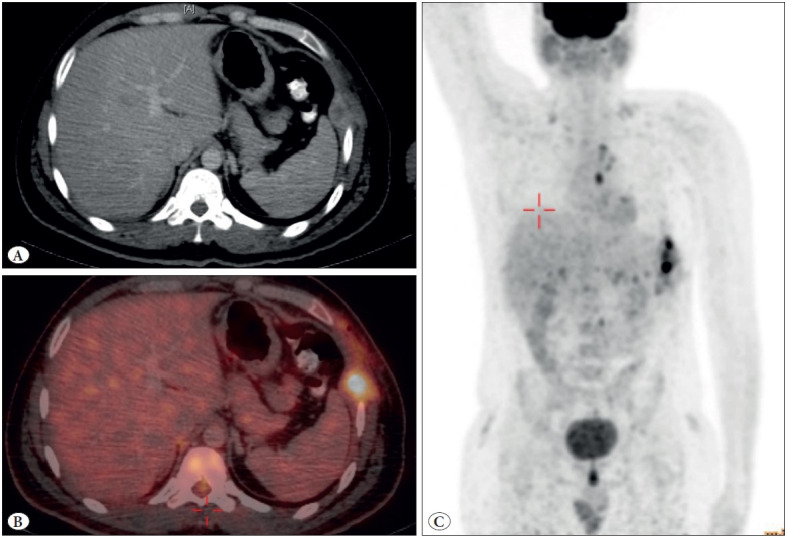
Fluorine-18 fluorodeoxyglucose Positron emission tomography/ Contrast enhanced computed tomography scan images showing an **A)** ill defined lobulated hypodense soft tissue lesion with peripheral enhancement in the left chest wall at the post-operative site, **B)** which is metabolically active. The lesion extends along the 7th and 8th intercostal spaces. **C)** Few metabolically active mediastinal nodules are also noted.

Positron emission tomography (PET) scan revealed an ill-defined hypodense soft tissue lesion with peripheral enhancement at the post-operative site, measuring approximately 5 x 3 cm, with a maximum SUV of 15.48 Mev, suggestive of either residual malignancy or Koch’s etiology (in view of the past history of tuberculosis). Few mediastinal pleural nodules were also noted at the D3/D4, D4/D5 and D6 levels. However, uptake on PET scan was not significant (Figure 1). Based on the imaging findings, and as the residual lesion was resectable, the patient was re-explored and en-bloc excision of the tumor with a wedge resection of the left lower lobe and 7th-10th rib was performed. However, complete R0 excision could not be achieved, because intra-operatively multiple small mediastinal and diaphragmatic pleural nodules were identified, and confirmed to be positive for malignancy on intraoperative frozen section.

**Figure 2:**
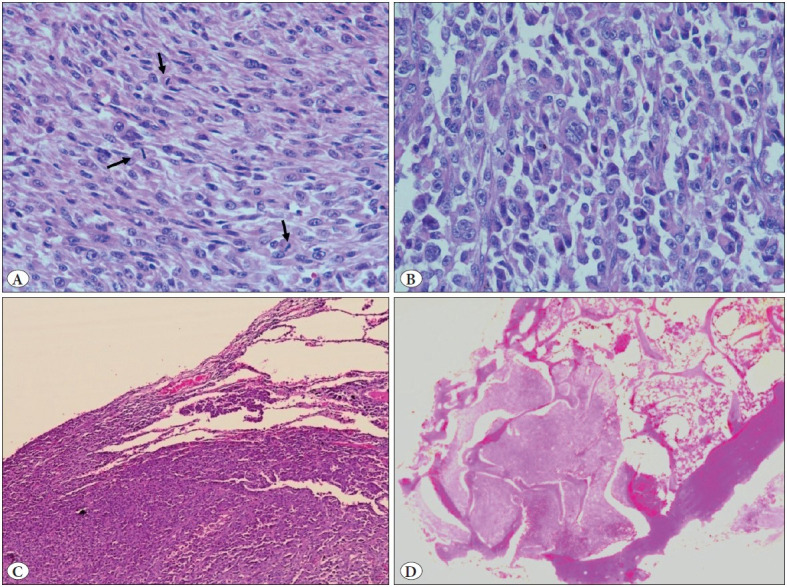
**A)** Spindle shaped tumor cells in sheets with vesicular nuclei and prominent nucleoli. Frequent mitosis is seen (arrows) (H&E; x400). **B)** Other areas showing dissociated epithelioid to rhabdoid tumor cells with abundant cytoplasm and eccentric, pleomorphic vesicular nuclei with prominent nucleoli. Multinucleation is noted (H&E; x400). **C)** Tumor infiltration in the lung (H&E; x100). **D)** Tumor invasion in underlying bones (rib) (H&E; x40).

**Figure 3:**
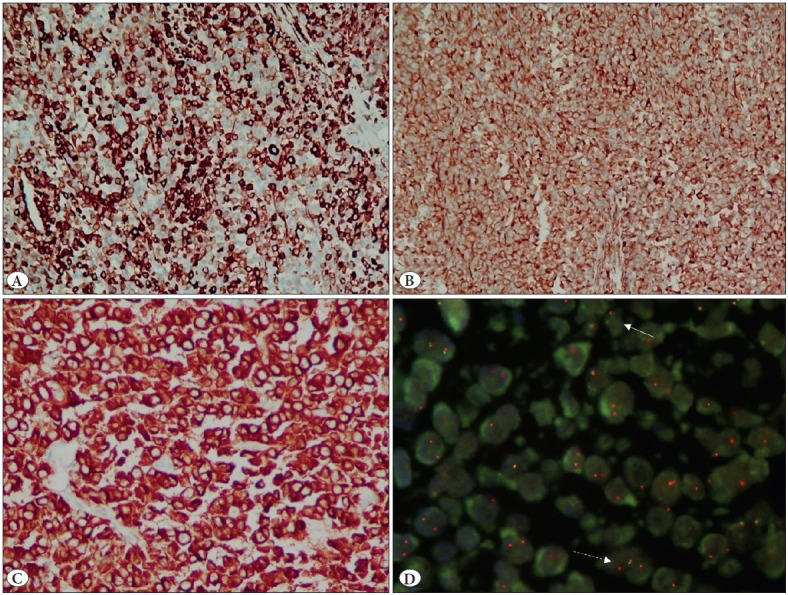
**A)** Strong immunopositivity for CK7 in the majority of the tumor cells (IHC; x100). **B)** Strong diffuse immunopositivity for Vimentin (IHC; x100). **C)** Strong, diffuse, granular immunopositivity for ALK-1 (IHC; x200). **D)** Break apart Fluorescence In-situ hybridization (FISH) for ALK showing split signal (white arrow) and loss of green signal (broken arrow). More than 15% of the tumor cells showing splits and loss of green signals was considered as positive for ALK gene rearrangement.

Histopathological examination revealed a tumor with a similar morphology to that of the prior excision, albeit with more spindled and sarcomatous appearing areas and numerous lymphovascular emboli (Figure 2). The tumour was also seen to involve the lung parenchyma and pleura and showed mediastinal lymph node metastasis. The tumor was additionally strongly positive for vimentin, while negative for estrogen receptor, Napsin A, and p63 (Figure 3). The special stain for mucin (mucicarmine) was negative.

Based on the imaging finding, spindle cell histomorphology and IHC results, a diagnosis of PSC of the spindle cell carcinoma type was made.

Further treatment options, including adjuvant radiotherapy and/or chemotherapy were discussed in a multidisciplinary clinic, in view of residual disease (R2 resection), with metastatic mediastinal lymphadenopathy. The patient was started on palliative chemotherapy (paclitaxel and carboplatin) and molecular profiling of the tumor was initiated. The tumor showed strong diffuse granular cytoplasmic positivity for ALK-1 Amplification IHC using the ventana D5F3 antibody clone. Subsequently, ALK-1 gene rearrangement was confirmed, using break apart fluorescence in situ hybridization (FISH) probes (Figure 3). The tumour was negative for other biomarkers i.e. EGFR mutations, ROS1 rearrangements and MET amplification. The tumor cells showed focal positivity of weak intensity for PDL1 with the Ventana SP263 antibody clone.

Considering ALK1 positivity, the patient was switched to Crizotinib. However he developed transaminitis and Crizotinib had to be discontinued after only six days of therapy. The patient eventually developed lower back pain, with difficulty in walking. An MRI scan revealed extradural soft tissue metastatic lesions at the D4-5 and D8-9 regions, for which the patient received palliative radiotherapy. However, his pain gradually worsened, with increasing sensorimotor deficit. Hence, he was restarted on Crizotinib with monitoring of liver function. Nevertheless, the patient’s general condition kept on deteriorating over the next few days. He developed abdominal distension and respiratory distress and succumbed to cardiac arrest despite all efforts.

## DISCUSSION

PSC is a heterogeneous group of rare and aggressive lung tumors, with unclear histogenesis, that accounts for 0.1%-0.4% of all lung cancers (1). PSC typically occurs in older, heavily smoking men and has a predilection for upper lobe involvement (1,2). The classification of sarcomatoid carcinomas of the lung has posed significant challenges over the years, due to their rare occurrence and heterogeneous histology. According to the most recent 2015 World Health Organization (WHO) classification of lung tumours, PSC is not a single entity but has five subgroups, which include: pleomorphic carcinoma, spindle cell carcinoma, giant cell carcinoma, carcinosarcoma, and pulmonary blastoma (1). Early surgery with post-operative adjuvant chemotherapy remains the preferred treatment. However, the prognosis remains poor, even in early stage disease (6,7).

We have documented here an extremely challenging case of PSC, unravelling the difficulties faced for establishing the diagnosis, as well as during treatment. The demographic profile was unusually different, as the patient was young (33 years old) and a non-smoker. Further, due to presence of coexisting tuberculosis in this patient, the possibility of malignancy was not considered initially, both clinically as well radiologically.

A diagnosis of PSC may be suspected on small biopsy or cytology, but commonly requires surgical resection to reach a conclusive definition (1). PSCs, and especially spindle cell carcinoma, are usually entirely composed of spindle-shaped cells without differentiated carcinomatous elements. The key role of IHC is to distinguish sarcomatoid carcinomas from sarcomatoid mesothelioma, primary or metastatic sarcomas, and metastatic sarcomatoid carcinoma (e.g., renal), as well as to exclude mimics, such as metastatic melanoma (1,7,8). Although, cytokeratins have significant diagnostic value in sarcomatoid carcinomas, staining is often focal and weak, and in some cases it can even be completely negative. Hence, it can be useful to utilize a panel of cytokeratins in suspected PSC. The markers of glandular and squamous differentiation, i.e., TTF1 and p40, respectively, can be negative as observed in the current case, but these markers should always be used as focal positivity can be seen in a subset of cases (7,8). In this case, distinction from sarcomatoid mesothelioma was difficult, considering the clinical presentation and histomorphological findings. Further, IHC results for specific differentiation markers may be negative in both tumor types. Sarcomatoid mesotheliomas are commonly negative or only weakly or focally positive for mesothelial markers. Pathologists should also keep in mind that mesothelial markers may be aberrantly expressed in PSC. BAP-1 negativity and GATA binding protein 3 (GATA3) positivity have been recently described to be very helpful in ruling out PSC and supporting the diagnosis of sarcomatoid mesothelioma (8).

Finally, distinction of sarcomatoid carcinoma from primary or metastatic sarcoma can be equally problematic. Just as sarcomatoid carcinomas may be virtually negative for keratins, some high-grade sarcomas are known to express keratins, usually weakly and focally. Thus, focal labeling for keratins should not be used as the sole criterion supporting the diagnosis of sarcomatoid carcinoma over sarcoma, and conversely, the lack of detectable keratins, particularly in a small sample, does not favor sarcoma over sarcomatoid carcinoma (8). Hence, a careful evaluation of clinicopathological, IHC and molecular findings has been advised to render the correct final diagnosis.

The molecular characterization of lung adenocarcinoma has revolutionized the treatment of lung cancer with the discovery of targetable genetic alterations such as EGFR mutations and ALK gene rearrangements (3,4). Specific targeted agents like gefitinib, afatinib, osimertinib (for EGFR mutated tumors) and crizotinib/ceritinib (for ALK rearranged lung adenocarcinomas) have been approved by the Food and Drug Administration (FDA) for clinical use (3).

Due to the rarity of PSC, the molecular profile has been barely characterized. The unresolved diagnostic, prognostic and therapeutic issues underline the urgent need to better define the molecular profiles of these tumors in order to identify new potential markers (5,7). Different studies have reported that a significant percentage of PSC cases may present gene alterations similar to other NSCLC types and alteration in tumor protein p53 gene (TP53) can be found in approximately 70% of PSC cases. In addition, KRAS (30-40% of patients) and MET genes (13-20%) are the most common driver oncogene mutations noted in PSC (7). However, targetable mutations in PSC are less frequent than in NSCLC with adenocarcinoma histology, with EGFR mutations reported in 8-22% cases in various studies (7,9–11). The efficacy of EGFR tyrosine kinase inhibitors (TKI) in PSC has also varied between studies, and was inferior when compared to adenocarcinoma (7).

Of note, none of these large studies described ALK gene rearrangement in PSC. A study of head and neck sarcomatoid carcinoma demonstrated ALK gene rearrangement in two out of 10 cases (20%), one of whom was administered Crizotinib and showed symptom improvement and disease stabilization for four months (12). The first case of ALK-positive PSC in an elderly non-smoker female was reported by Ali et al., in a metachronous second lung primary after resection of a lung adenocarcinoma, which also showed ALK-1 gene rearrangement (13). Since then, only handful of reports of ALK rearrangements were reported in PSC and the effects of targeted therapies against this mutation have been shown to be varied, but mostly with ineffective or partial responses (13–16).

On molecular analysis of 33 PSC cases, Terra et al. found ALK gene rearrangement in a single case (3%), similar to the reported frequency in adenocarcinoma (5). The patient, in contrast to ours, was a 58-year-old female with an eight pack-year smoking history. The first report of ALK-rearranged advanced PSC treated by Crizotinib was by Murakami et al. in 2015, but the response was transient (14). Karim et al. demonstrated EML4-ALK translocations in two patients out of 25 cases of PSC. One of the patients received Crizotinib and had the longest overall survival compared to the other patients in the study cohort (15).

Crizotinib was started in view of advanced stage in the index case, as this PSC revealed ALK1 gene rearrangement. However, it had to be temporarily stopped due to drug toxicity, and the patient succumbed to death within a very short time. Hence, the clinical impact of identifying this targetable mutation could not be established in our case.

In addition, MET amplification and mutations have also been described in PSC (17). Considering that Crizotinib targets both ALK and MET mutations, this agent might play an important role in treating patients with PSC. Additional studies are required to clearly determine the efficacy of molecular targeted therapies against PSC.

Recently, immunotherapy has also demonstrated encouraging results in the treatment of NSCLC. Although PDL-1 expression was noted in only 5% of the tumor cells in the present case, high PDL-1 expression (53% and 69.2%) has been recorded by some researchers (18,19). This suggests that PSC expresses greater levels of PD-L1 and it may represent a potential therapeutic target for PSC in the future.

The co-existence of pulmonary tuberculosis has been reported in 0.7% of lung cancer cases (20). The association of tuberculosis with PSC has not been reported in any of the prior published reports in the literature. Significant overlap in the clinical presentation and radiological features was reported between tuberculosis and lung cancer (21,22). Chandra et al. had reported that, out of 123 lung cancer patients, 23 (17%) had been initially labelled and treated as tuberculosis, resulting in a significant delay in the diagnosis of malignancy (mean difference of 65.6 days). India being an endemic country for tuberculosis, starting of AKT for any suspicious opacities on chest radiographs without proper evaluation was the main reason assigned for the delay in lung cancer diagnosis and treatment. Thus, in patients with already confirmed active tuberculosis, presence of lung cancer is rarely suspected, with consequent delay in diagnosis resulting in advanced stage at presentation as happened in the index case (23). According to Wu et al., an occult lung cancer may reactivate the tuberculosis by lowering the patient’s immunity in endemic countries where a significant proportion of the population is infected with latent tuberculosis. Conversely, chronic inflammation due to pulmonary tuberculosis itself may cause multistep transformation of cells, leading to lung cancer (24).

Although, our patient initially benefitted from AKT, the malignancy was clinically and radiologically masked, which had resulted in a delayed diagnosis, with mortal consequences. Further, he could not tolerate the Crizotinib well, which may be attributed to a possible drug interaction with AKT. Hence, this case highlights the difficulties encountered in diagnosing and treating a case of PSC with co-existing tuberculosis.

To conclude, we report here a rare case of ALK1 gene rearranged PSC, in a young non-smoker male, which was camouflaged by co-existent tuberculosis. We recommend that comprehensive molecular analysis, especially for ALK1, should be carried out in all PSCs to gather robust data regarding the molecular biology of this rare tumor. Hopefully, this will open up new therapeutic options for patients with this deadly disease in the future.

## CONFLICT of INTEREST

The authors declare no conflict of interest

## FUNDING

This research received no specific grant from any funding agency in the public, commercial, or not-for-profit sectors.
